# An evaluation of outpatient satisfaction based on the national standard questionnaire: a satisfaction survey conducted in a tertiary hospital in Shenyang, China

**DOI:** 10.3389/fpubh.2024.1348426

**Published:** 2024-05-09

**Authors:** Zhou Xintong, Xin Tao, Wang Shuying, K. A. T. M. Ehsanul Huq, Gao Huiying, Moriyama Michiko

**Affiliations:** ^1^Department of Hospital Infection Management, Shenyang the Fourth People’s Hospital, Shenyang, China; ^2^Graduate School of Biomedical and Health Sciences, Division of Integrated Health Sciences, Hiroshima University, Hiroshima, Japan; ^3^Department of Information, Shenyang the Fourth People’s Hospital, Shenyang, China; ^4^Department of Doctor-patient Communication, Shenyang the Fourth People’s Hospital, Shenyang, China

**Keywords:** patient satisfaction, healthcare survey, doctor–patient relationship, environment, factor analysis

## Abstract

**Background:**

Patient satisfaction survey serves as a pivotal tool in evaluating the quality of healthcare services. China’s nationwide standard patient satisfaction measurement tool was introduced in 2019. This study aimed to assess the model fit of the national standard outpatient satisfaction questionnaire in a tertiary hospital and evaluate the outpatient satisfaction levels using this tool.

**Method:**

A cross-sectional survey using the national outpatient satisfaction questionnaire was conducted via message links to all hospital outpatients who registered between April and July 2022. The data collected underwent descriptive analysis, comparative analysis, and confirmatory factor analysis (CFA).

**Results:**

A total of 6,012 valid responses were received and analyzed during this period, with 52.9% of the participants being women. The confirmatory factor analysis (CFA) model showed a good fit and identified doctor communication as having a positive effect and environmental factors as having a negative effect on outpatients’ satisfaction, with standardized regression weights of 0.46 and 0.42, respectively. Despite the remarkably high satisfaction levels, patients’ recommendation for using the services of this hospital surpassed the overall evaluation and total satisfaction scores.

**Conclusion:**

A disparity was identified between the expectations and real experiences of outpatients, leading to some extent of dissatisfaction. To enhance satisfaction levels, the hospital should improve the communication skills of all clinical staff, simplify the environment layout for first-time visitors, and manage patient overloads.

## 1 Introduction

Patient satisfaction survey serves as a pivotal tool in evaluating the quality of healthcare services (1, 2). Healthcare institutions can address issues and areas of improvement by collecting feedback from patients (3), enabling targeted enhancements to elevate the overall quality of healthcare services (4, 5). Additionally, this survey creates a platform for patients to voice their opinions and offer suggestions, fostering positive communication between patients and healthcare providers (6). This dynamic fosters the provision of positive doctor–patient relationships (7).

Since 2000, global research has significantly advanced the understanding and measurement of patient satisfaction (8). The United States introduced a Hospital Consumer Assessment of Healthcare Providers and Systems (HCAHPS) survey in 2006, which became the national standard for assessing patient satisfaction (9). England utilizes the General Practitioner Patient Survey (GPPS) authorized by the National Health Service (NHS) since 2009 (10), while Japan has conducted nationwide satisfaction surveys since the 1980s, employing tools such as the Japanese version of Primary Care Assessment Tool (JPCAT) (11) and HCAHPS (Japanese version) (12).

The development of patient satisfaction surveys in China is rather complex. The first report in 1993 had a conceptual bias about the patient satisfaction survey, which led to a prolonged confusion between “patient satisfaction” and “medical ethics” for over two decades (13–15). Recognizing this issue in 2015, the Chinese government launched the “China Healthcare Improvement Initiative (CHII)” and conducted the China National Patient Survey to rectify misunderstandings and enhance the quality of medical services (16, 17). In 2019, the National Health Commission of China introduced a nationwide satisfaction questionnaire, defining satisfaction as the disparity between patient expectations and actual practices (18–22). The first nationwide survey based on this developed standardized questionnaire was conducted in the same year (23).

Previous nationwide surveys on patient satisfaction in China primarily yielded broad outcomes, encompassing overall satisfaction and hospital recommendations throughout various regions of the entire country (16, 23–25). However, this nationwide survey did not provide individual feedback to the participating healthcare institutions, resulting in independent healthcare facilities being unaware of their own outpatient satisfaction levels. On the other hand, few healthcare facilities in China have conducted their own satisfaction surveys independently using the national version questionnaire, with one study focusing solely on female patients (18). Moreover, considering the significant regional variations in China and substantial differences in the quality of healthcare institutions at various levels, the adaptability of the nationwide satisfaction survey within individual healthcare facilities requires further discussion.

Hence, it is necessary to implement the national version questionnaire in a satisfaction survey conducted by an independent medical facility for two primary reasons: (1) to validate the suitability and adaptability of this standardized questionnaire and (2) to evaluate the satisfaction levels of individual healthcare facility using this standardized measurement and address main aspects for improvement.

To the best of our knowledge, this is the first study to validate the national satisfaction survey questionnaire and evaluate outpatient satisfaction levels in a tertiary hospital in China. The findings of the study will allow identifying the key pitfalls for the improvement of healthcare facilities, thereby enhancing the quality of medical services.

## 2 Method

### 2.1 Study design and setting

This is a cross-sectional survey using the national outpatient satisfaction questionnaire conducted in a tertiary general hospital (highest level hospital) with a specialty in ophthalmology in Shenyang, China. In this study, we collected data and used secondary data from the outpatients who attended the hospital. The hospital comprises 1,650 beds, with a staff of over 2,400 and an annual outpatient accounting of approximately 1,000,000. Within the hospital, the ophthalmology department has 315 beds, 380 staff members, and an annual outpatient range of 500,000 to 600,000. Adhering to the STROBE guidelines and reporting system (26), the study explores the outpatient experience in the context of the hospital’s standard process, where patients in China have the flexibility to access healthcare services without prior reservations. The standard outpatient process includes entering the hospital, choosing a department and doctor, registering, waiting for a face-to-face consultation, undergoing prescribed examinations, returning to the same doctor with test results, receiving a diagnosis and prescription, and obtaining medication from the outpatient pharmacy or other sources.

### 2.2 Participants and study procedures

The patient satisfaction survey at this hospital has been conducted through a professional patient satisfaction survey system since 2020. The system was adapted using the national satisfaction questionnaire from early 2022. The national outpatient satisfaction questionnaire used in this study was distributed to all registered patients via text message links from April to July 2022. Patients voluntarily decided whether to participate in the survey. At the beginning of the questionnaire, patients were provided with information about the purpose of the survey and the use of related information, and it was stated that participating in the survey implied consent to the use of survey information.

### 2.3 Measuring tool

The satisfaction questionnaire for outpatients was published by the National Health Commission of China as the measurement for the hospital satisfaction survey of the *“National tertiary public hospital performance appraisal operation manual (2022)”* (21). The questionnaire was based on the Chinese version of the HCAHPS (16). After the required modifications based on China’s national conditions were made, the questionnaire was tested for validity and reliability (Cronbach’s *α* = 0.935; *χ*^2^/*df* = 2.958, GFI = 0.974, AGFI = 0.955, RMR = 0.009, RMSEA = 0.044) (18).

The outpatient satisfaction questionnaire comprises a satisfaction survey consisting of six dimensions: “convenience” (2 items), “registration communication” (2 items), “doctor communication” (3 items), “nurse communication” (3 items), “environment and layout” (4 items), and “response of needs” (2 items). It also includes two general satisfaction indicators, namely, “patient’s overall evaluation of this hospital” (overall evaluation) and “recommendation of this hospital to others” (recommendation level). Second, socio-demographic questions incorporating seven items: “registration methods,” “registered department,” “type of registration,” “gender,” “age,” “educational background,” and “payment method” (described in Supplementary Table S1).

### 2.4 Statistical analysis

#### 2.4.1 Data preparation

A total of 7,789 patients responded out of 402,964 registered outpatients (1.93% responded); a total of 1,777 invalid replies out of 7,789 (22.8%) were excluded due to duplicate or contradictory answers (consistency bias); and finally, 6,012 valid replies were used for analyses. Sum scores of satisfaction questions were calculated for each reply.

#### 2.4.2 Data analysis

SPSS Statistics 28.0 and SPSS Amos 28.0 (IBM) were used in this study for descriptive analysis, comparative analysis, and confirmatory factor analysis (CFA). First, the sum score of each reply was calculated for normal distribution tests. Then, demographic questions were described as frequencies and constituent ratios. The month was considered as a categorical variable. Then, each satisfaction question was described as frequencies and constituent ratios and also calculated for means and standard deviations (SD). Continuous variables were described using minimum and maximum values, as well as means and standard deviations (SD). To verify the model fit of the questionnaire in an independent medical facility, the data were tested for reliability and model fit. The goodness-of-fit indices and their ranges include the following: the chi-square/df ratio (*χ*^2^/*df*) < 3, the goodness-of-fit index (GFI) > 0.9, the adjusted goodness-of-fit index (AGFI) > 0.9, the parsimony goodness-of-fit index (PGFI) > 0.9, the normed fit index (NFI) > 0.9, the relative fit index (RFI) > 0.9, the incremental fit index (IFI) > 0.9, the Tucker–Lewis index (TLI) > 0.9, the comparative fit index (CFI) > 0.9, the standardized root mean square residual (SRMR) < 0.05, and the root mean square error of approximation (RMSEA) < 0.05. The differences in general satisfaction levels among outpatient demographic characteristics were assessed using a *t*-test or analysis of variance. To compare the results of the overall evaluation, recommendation level, and total satisfaction score, their means were converted into corresponding percentages. Finally, the area’s most in need of improvement were allocated by comparing the items with the lowest means with other factors. The significance level was set at <0.05. *p*-values exceeding 0.05 are not listed or marked in the subsequent content.

## 3 Results

### 3.1 Socio-demographic characteristics of patients

A total of 7,789 patients responded out of 402,964 registered outpatients (1.93% response rate). Table 1 describes the socio-demographic characteristics of patients. In April, due to the impact of the “lockdown” policy, the number of responses collected was half of those in other months. Participants predominantly opted for “Appointment” for registration (57.7%). Ophthalmology department patients constituted 38.0% of the participants, while 17.2% solely registered for COVID-19 PCR tests. A total of 60.4% opted for normal registration. Women (52.9%) and those in their 40s and 50s (30.5%) were slightly more common, and 67.0% of the participants had undergraduate or graduate degrees, which is higher than the Chinese population average. In this survey, 58.1% of outpatient participants opted for self-payment.

**Table 1:** Socio-demographic characteristics of the participants.

Socio-demographics	Count	Percent	Socio-demographics	Count	Percent
Month (data collected)			Age (Group)		
April	862	14.3%	< 20	728	12.1%
May	1907	31.7%	20 ~ 39	1,622	27.0%
June	1837	30.6%	40 ~ 59	1835	30.5%
July	1,406	23.4%	60 ~ 79	1,663	27.7%
Registration method			≥ 80	164	2.7%
Window	2,126	35.4%	Education background		
Appointment	3,468	57.7%	Middle school or lower	771	12.8%
Self-service machine	274	4.6%	High school	1,209	20.1%
Others	144	2.4%	Undergraduate	3,508	58.3%
Department registered			Graduate	524	8.7%
Internal medicine	1,638	27.2%	Payment method		
Surgical	618	10.3%	Free medical insurance	735	12.2%
Ophthalmology	2,286	38.0%	Urban medical insurance	1,672	27.8%
Obstetric and pediatric	358	6.0%	Rural medical insurance	113	1.9%
COVID-19 PCR	1,035	17.2%	Self-payment	3,492	58.1%
Others	77	1.3%	Waiting time		
Type of registration			≤ 15 min	2015	95%
Normal	3,631	60.4%	> 15 min	100	5%
Special	2,381	39.6%	Missing	3,897	—
Gender					
Men	2,829	47.1%			
Women	3,183	52.9%			

Regarding “waiting time in the queue before registration” (waiting time), it was reported as 1 min to 180 min; however, over 95% were less than 15 min.

### 3.2 Model fit analysis: confirmatory factor analysis

The total score of this questionnaire ranges from 10 to 74. Valid responses (6,012) fit a normal distribution with a mean (SD) of 56.8 (9.75), a minimum of 13 and a maximum of 73.

#### 3.2.1 Validity and reliability

The Kaiser–Meyer–Olkin (KMO) measure for the questionnaire except socio-demographic data yielded a high value of 0.933. Bartlett’s test returned a statistically significant result (*p* < 0.001). The internal consistency was confirmed by Cronbach’s alpha coefficient of 0.772.

#### 3.2.2 Confirmatory factor analysis model fit

**Figure 1:**
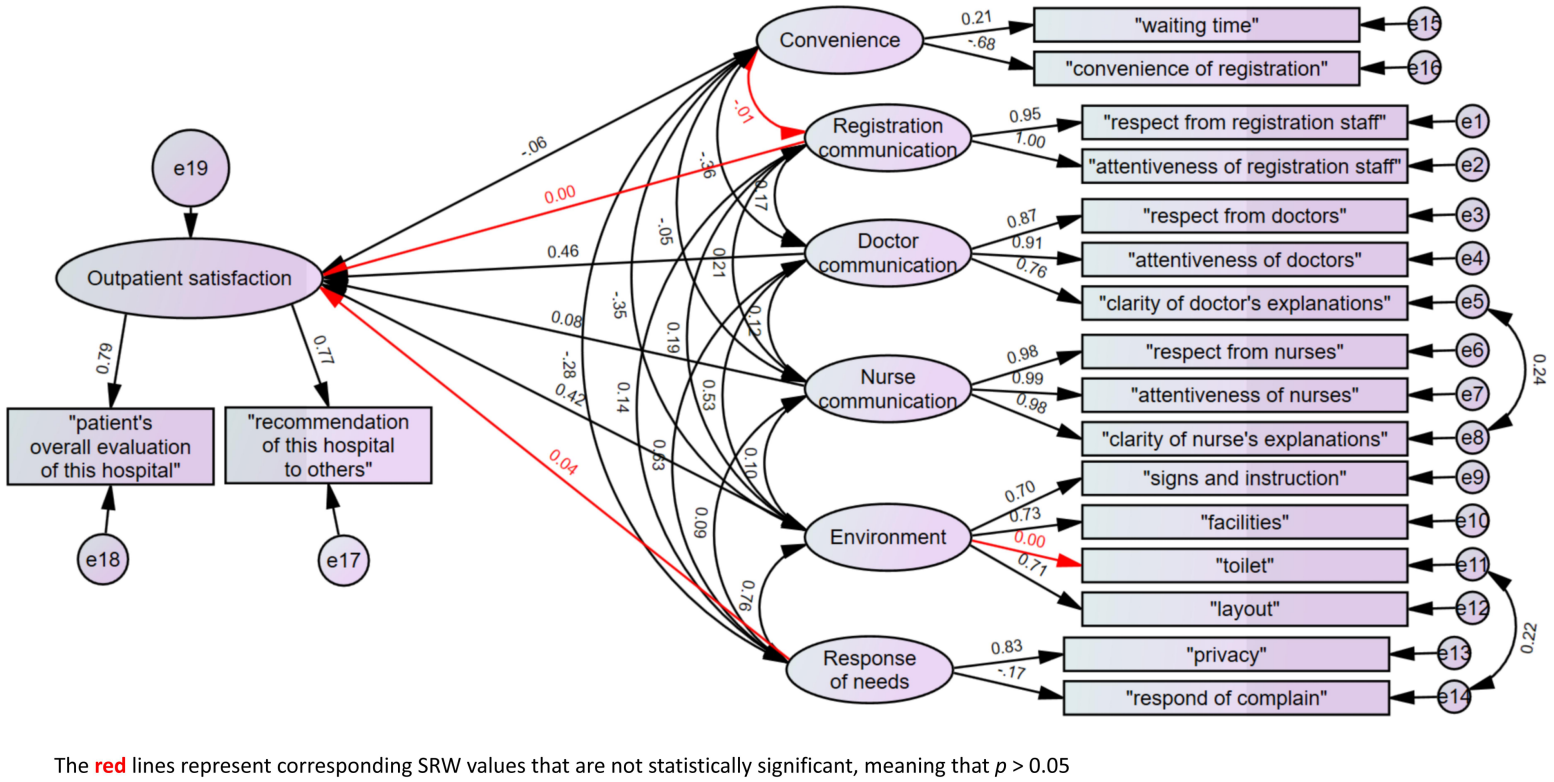
CFA model and standard regression weights (SRW) of outpatient satisfaction.

Figure 1 shows the model structure for estimating the various statistical relationships involved in outpatient satisfaction, and Table 2 demonstrates the results of indices. The model fit appears robust, as evidenced by NFI, RFI, IFI, TLI, CFI, GFI, and AGFI, all surpassing 0.9. The RMSEA value is below 0.05. This CFA model with standard regression weights (SRW) of outpatient satisfaction figured out two primary factors influencing outpatient satisfaction, which were “doctor communication” (0.46) and “environment” (0.42) (both, *p* < 0.001). Factors such as “convenience” (−0.06) and “nurse communication” (0.08) indicated minor contributions (both, *p* < 0.01), while “registration communication” (*p* = 0.975) and “response of needs” (*p* = 0.174) did not contribute significantly to outpatient satisfaction. Regarding the factor “environment,” “toilet” did not have statistical significance (*p* = 0.965). “Doctor communication” exhibited a strong correlation with both “environment” and “response of needs.” Simultaneously, the factor “response of needs” demonstrated a high correlation with the “environment” as well. The standard regression weights among them were 0.53, 0.63, and 0.76, respectively.

**Table 2:** Model fit indices.

χ^2^/df	14.652
GFI	0.970
AGFI	0.955
PGFI	0.636
NFI	0.979
RFI	0.971
IFI	0.980
TLI	0.973
CFI	0.980
SRMR	0.050
RMSEA	0.048

The modification indices suggested including covariance between errors in “clarity of doctor’s explanations” and “clarity of nurse’s explanations” as well as between “toilet” and “response of complaint.”

### 3.3 Satisfaction level of outpatients

The mean (SD) of “patient’s overall evaluation of this hospital” (overall evaluation) was 8.43 (1.97). Over 75% of the patients marked 8 or above, and more than 25% of patients scored 10 (full score). A similar key question asking “recommendation of this hospital to others” (recommendation level) marked 3.43/4 (full score; described in Supplementary Table S2).

**Figure 2:**
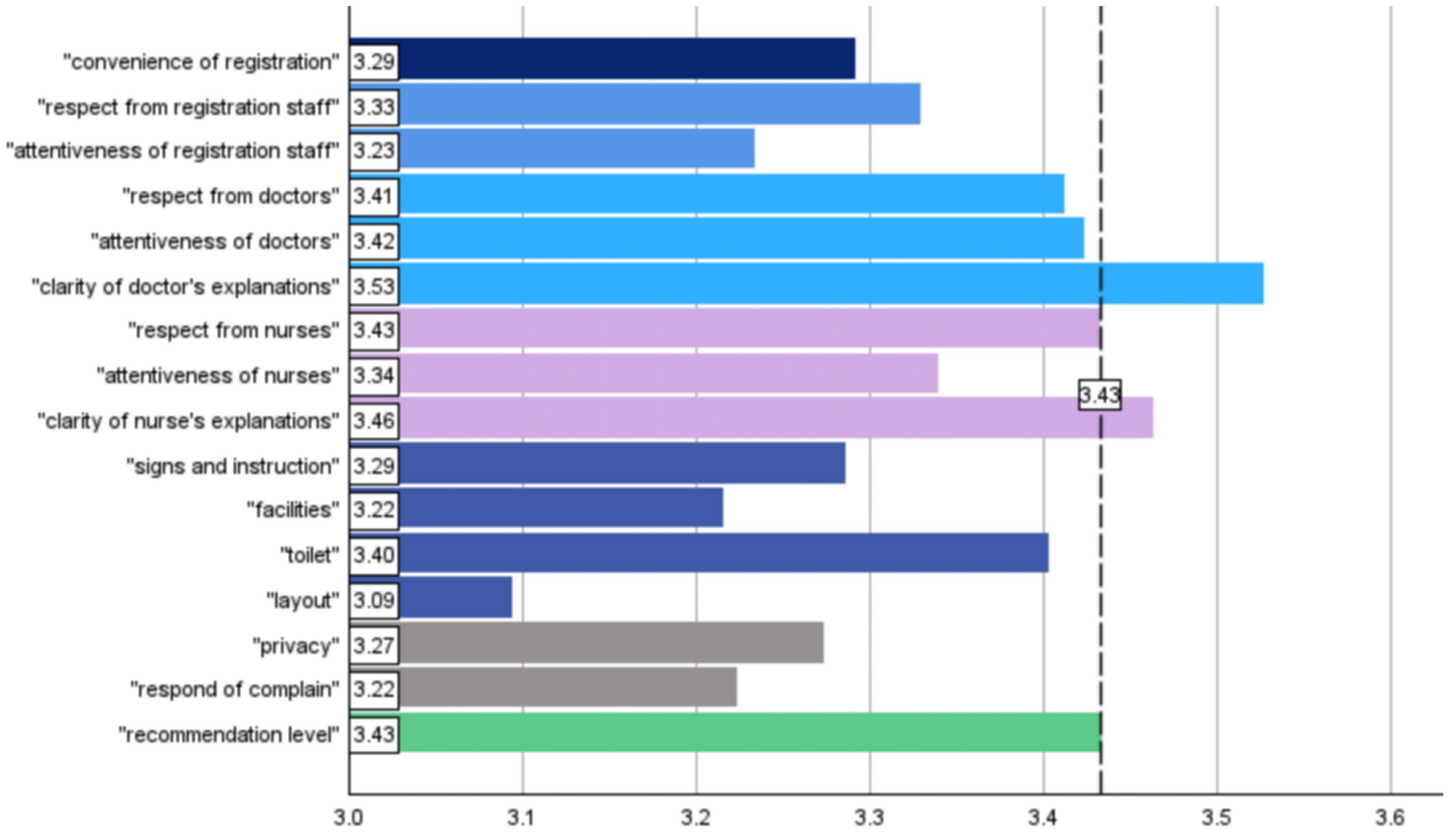
Adjusted means of satisfaction items.

The adjusted mean of satisfaction of individual questions is shown in Figure 2. When “recommendation level” (mean score = 3.43) was set as the reference value, “clarity of doctor’s explanations” (mean = 3.53) and “clarity of nurse’s explanations” (mean = 3.46) achieved higher means than the reference value. Contrary, “layout” from “environment” recorded the lowest mean of 3.09 (details of these satisfaction questions are listed in Supplementary Table S2).

### 3.4 Analyzing the influence of patient’s socio-demographics on general satisfaction level

“Overall evaluation,” “recommendation level,” and the “total score” of all questions, all categorized as general satisfaction indicators, were compared across socio-demographic characteristics of the patients (Table 3). Significant differences in all three general satisfaction indices were observed concerning the “month,” “department of registration,” “age (group),” and “payment method.” The highest satisfaction scores were observed in April, among those using the “window” registration, seeking services in the “internal medicine” department, being “over 80 years old,” and being covered by “free medical care,” all with statistically significant differences (all, *p <* 0.05).

**Table 3:** General satisfaction indicators grouped by demographics.

Values	“Overall evaluation”			“Recommendation level”			Total score		
	*M*	SD	*P*-value	*M*	SD	*P*-value	*M*	SD	*P*-value
Month									
April	8.70	1.92	<0.001	3.62	0.61	<0.001	58.34	9.25	<0.001
May	8.42	1.95		3.53	0.64		56.49	9.77	
June	8.36	2.00		3.52	0.66		56.58	9.82	
July	8.37	1.98		3.54	0.65		56.57	9.85	
Registration method									
Window	8.61	1.83	<0.001	3.57	0.61	0.066	59.60	8.46	<0.001
Appointment	8.32	2.02		3.52	0.66		55.09	10.00	
Self-service machine	8.41	2.21		3.57	0.66		56.11	10.30	
Others	8.41	2.05		3.57	0.61		58.04	10.68	
Department you registered									
Internal medicine	8.78	1.65	<0.001	3.59	0.60	<0.001	59.33	8.75	<0.001
Surgical	8.46	1.98		3.50	0.68		55.65	10.55	
Ophthalmology	8.30	1.97		3.54	0.65		55.93	9.37	
Obstetric and pediatric	8.41	1.93		3.46	0.61		56.27	9.62	
COVID-19 PCR	8.19	2.32		3.52	0.66		55.69	10.72	
Others	8.18	2.37		3.35	0.81		55.70	11.82	
Type of your registration									
Normal	8.44	1.96	0.403	3.53	0.64	0.054	56.61	9.83	0.066
Special	8.42	1.99		3.56	0.65		57.09	9.62	
Gender									
Men	8.41	1.99	0.371	3.53	0.66	0.146	57.07	10.03	0.042
Women	8.45	1.95		3.55	0.63		56.56	9.49	
Age (Group)									
< 20	8.15	2.07	<0.001	3.50	0.64	<0.001	55.06	9.61	<0.001
20 ~ 39	8.16	2.16		3.43	0.70		54.95	10.38	
40 ~ 59	8.55	1.86		3.59	0.61		57.31	9.63	
60 ~ 79	8.67	1.81		3.62	0.61		58.61	8.95	
≥ 80	8.59	1.82		3.62	0.56		58.91	8.57	
Education background									
Middle school or lower	8.49	2.16	0.419	3.64	0.60	<0.001	58.41	9.18	<0.001
High school	8.41	2.06		3.54	0.68		56.82	9.89	
Undergraduate	8.44	1.88		3.53	0.63		56.57	9.68	
Graduate	8.31	2.07		3.50	0.68		55.96	10.44	
Payment method									
Free medical insurance	8.86	1.57	<0.001	3.65	0.56	<0.001	60.64	8.18	<0.001
Urban medical insurance	8.63	1.73		3.59	0.59		58.66	8.79	
Rural medical insurance	8.28	2.46		3.61	0.60		59.13	10.75	
Self-payment	8.25	2.11		3.49	0.68		55.03	10.04	

### 3.5 Analyzing primary factors that affect patients’ general satisfaction levels

**Figure 3:**
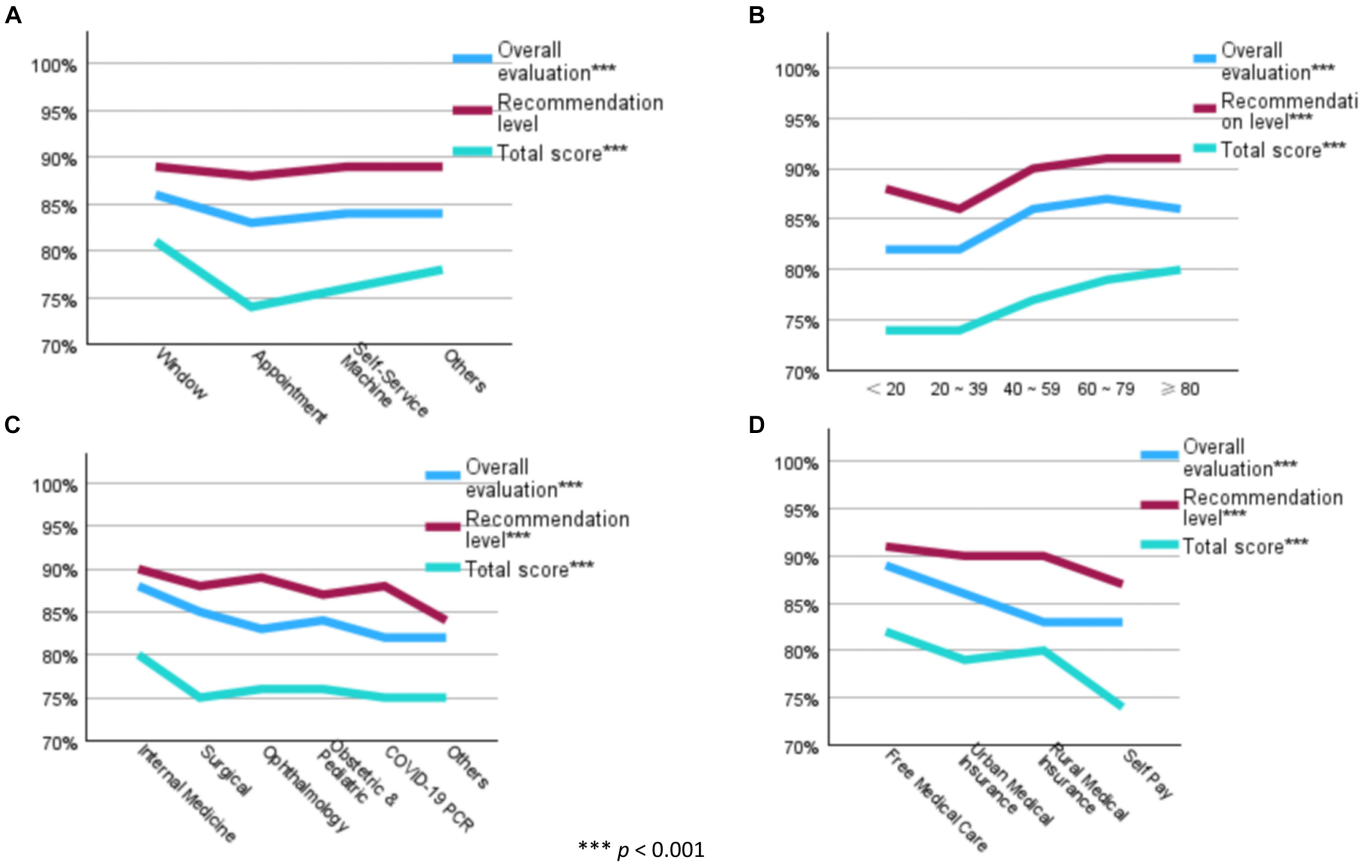
Corresponding percentage of the means of general satisfaction indicators. **(A)** Registration Method; **(B)** Age (Group); **(C)** Department you registered; **(D)** Payment method.

To find the gap among the three indices in each socio-demographic characteristic, each score has been transformed into its respective percentage. Figure 3 describes further analysis of the four factors that indicate statistical significance. “Appointment” and “younger age groups” scored lower satisfaction levels compared to other groups (both, *p* < 0.001). “Internal medicine” and “free medical care” scored higher than other groups (both, *p* < 0.001). Supplementary Table S3 reveals a consistent pattern where the “recommendation level” consistently surpassed both the “overall evaluation” and the “total score.”

### 3.6 Investigating influencing factors on primary factors

**Figure 4:**
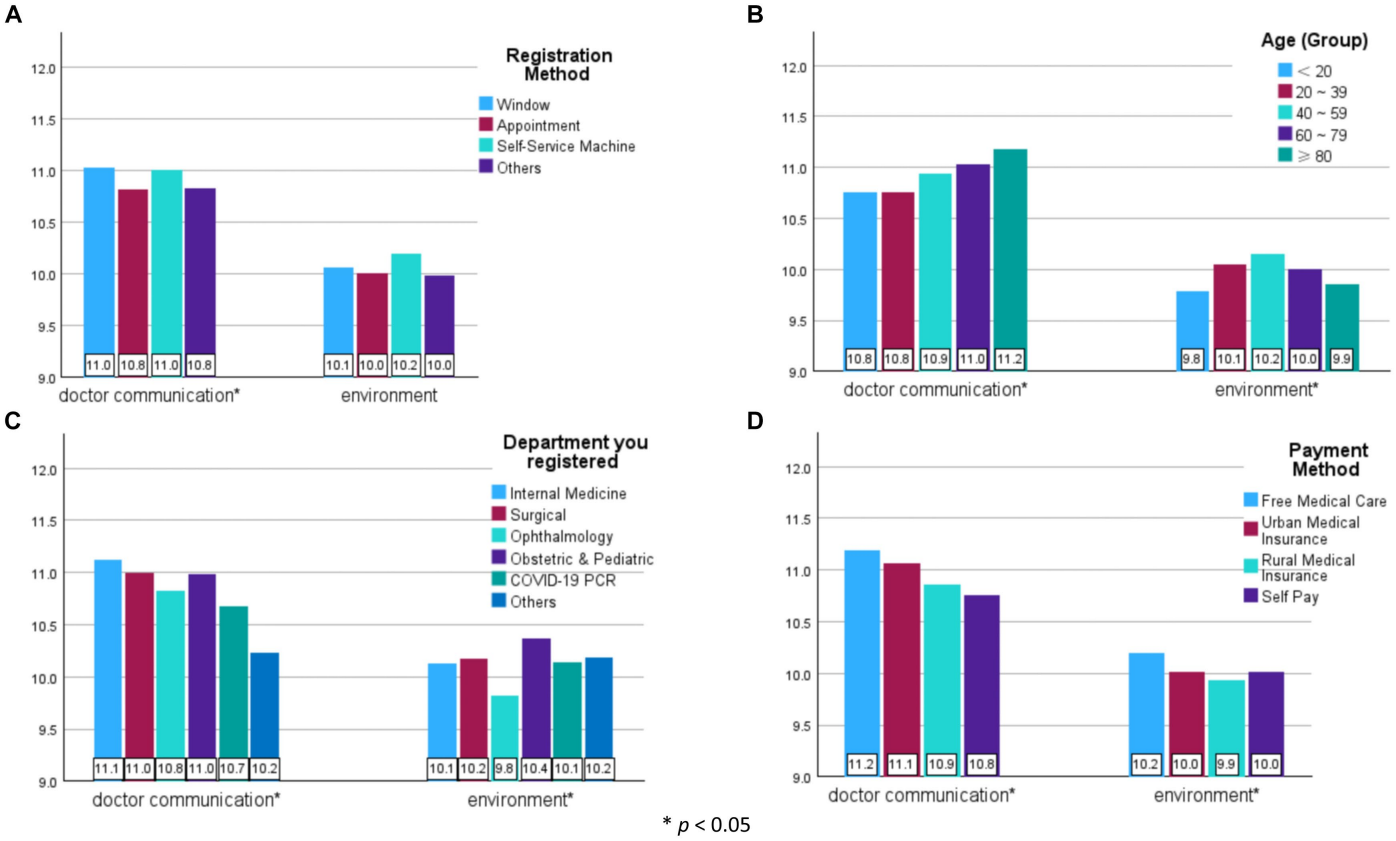
Comparison of the sum scores of the two primary factors. **(A)** Registration Method; **(B)** Age (Group); **(C)** Department you registered; (D) Payment method.

Two primary factors, “doctor communication” and “environment,” which contributed most to outpatient satisfaction levels, were further analyzed among “registration method,” “age (group),” “department registered,” and “payment method.” The attributes with the highest satisfaction score (mean) for “doctor communication” consisted of patients who registered at the “window,” used the “self-service machine,” were “over 80 years old,” visited the “internal medicine” department, and had “free medical care.” For “environment,” patients who registered at the “self-service machine,” were “aged 40–59,” visited the “obstetric & pediatric” department, and had “free medical care” scored the highest mean. In particular, patients who were “under 20 years old” and “over 80 years old,” visited the “ophthalmology” department and had “rural medical insurance” scored the lowest for the “environment” factor (all, *p* < 0.05; Figure 4).

## 4 Discussion

This marks the inaugural publication detailing the outcomes of a national satisfaction survey conducted at a tertiary hospital in China. Through rigorous examinations of validity and reliability, we ascertained that the survey consistently adhered to the established questionnaire structure. The comprehensive approach of the survey effectively identified six influencing factors in outpatient satisfaction: doctor communication, environment, registration method, age (group), department of registration, and payment method. This conclusion is supported by a substantial volume of outpatient data.

### 4.1 Questionnaire adaptability and its influences

The utilization of advanced “SMS questionnaire surveys” has resulted in the comprehensive sample in this study exhibiting favorable characteristics of normality, reliability, and validity. The CFA model demonstrated a good fit, despite a *χ*^2^/df ratio of 14.652, surpassing the conventional threshold of 3 (Table 2). It is crucial to note that, given the substantial sample size exceeding 6,000, this deviation may not necessarily undermine the overall model fit. Therefore, this result supported the original structure of this satisfaction questionnaire.

The CFA model (Figure 1) revealed two primary factors influencing outpatient satisfaction: doctor communication and environment (27, 28). These factors emerge as consistent contributors to satisfaction, aligning with results observed in both Chinese and international contexts (29–35).

### 4.2 Satisfaction level and influencing factors

This study reveals that the hospital has exceeded both national and northeast regional averages in scores for overall assessment and recommendation level (16). The significant factor contributing to this achievement is the outstanding performance of the hospital in the realm of doctor communication, particularly in the facet of clarity of doctor’s explanations, where it achieved the highest score (Figure 2). A wealth of research consistently supports the idea that thorough and effective communication by healthcare providers plays a pivotal role in significantly enhancing patient satisfaction levels (6, 30, 32, 36).

The study pinpointed patient overload as a critical factor influencing satisfaction. The significant surge in patients contributes to a congested clinical environment (37), resulting in prolonged waiting times at various stages and a diminished duration of patients’ consultations with physicians (38), adversely affecting their satisfaction levels (39–41). The ophthalmology department at this hospital faced significant patient overload, evident in low ratings for the environment factor and moderately rated doctor communication, impacting overall satisfaction (Figure 3C). Patients using the appointment registration displayed the lowest satisfaction (Figure 3A), likely due to appointment non-adherence issues from high patient volume. The inclusion of the toilet item from the environment factor, linked to the response of needs (Figure 1), highlights insufficient toilet stalls, potentially stemming from overwhelming patient volume. In contrast, April achieved the highest satisfaction due to minimal patient count during the COVID-19 situation and the “lockdown” policy in China (42) (Supplementary Table S3A).

Patient satisfaction is influenced by factors such as gender (43–45), age (25, 46), and educational background (47) (Table 3), aligning with similar patterns observed in other research studies.

### 4.3 The impact of unique Chinese factors on patient satisfaction

#### 4.3.1 Registration method

In China, registration commonly occurs through face-to-face visits at hospital windows, but technological advancements have introduced alternatives such as online appointments through websites or mobile apps. Self-service machine registration also serves as an effective alternative, usually accompanied by staff guiding patients on the usage of the machine. Apart from these three methods, there are also other registration methods, such as phone appointments.

This study reveals that patients who registered at the window expressed the highest satisfaction level. Additionally, those using window or self-service machine registrations tend to rate doctor communication more positively (Figure 4, Registration Method). This suggests that these two registration methods may provide more communication opportunities compared to online appointments and others, thus enhancing satisfaction levels (48).

#### 4.3.2 Payment method

China has three primary government medical insurance programs: (1) Free medical insurance for public service people, covering all medical expenses; (2) Urban medical insurance for urban inhabitants, reimbursing a significant portion of medical costs, with higher coverage rates for residents; and (3) Rural medical insurance for rural inhabitants, covering most medical expenses but excluding transportation and accommodation. Other payment methods not covered by these programs are collectively referred to as “self-payment.”

As patient groups shift from free medical insurance to self-payment, their financial burden increases, resulting in a decline in overall satisfaction, especially regarding doctor communication (Figure 4, Payment Method). Previous research indicates that as economic burdens rise, information needs by patients increase (49). Hence, despite receiving equivalent information from doctors, self-payment patients may experience a perceived information deficit caused by the increasing demand for services commensurate with more payment by themselves, leading to dissatisfaction. This finding aligns with previous studies (50, 51).

### 4.4 Gaps between patients’ expectations and actual experience

When patients choose a medical institution, recommendations from others play a crucial role. This consistent “reputation influence” reflects profound acknowledgment, amplifying patients’ expectations of the hospital (52, 53). In this study, the recommendation level consistently holds the highest position, indicating that patients’ acknowledgment and expectations are extremely high (54). Meanwhile, the total score, representing patients’ actual experiences, consistently ranks at the bottom among the three indices (Figure 3; Supplementary Table S3). This underscores a disparity between patients’ high expectations and their actual experiences, resulting in a lower satisfaction level (55). This finding elucidates the phenomenon of patients being satisfied but also experiencing some level of dissatisfaction with medical facilities in China, aligning with previous research findings (56, 57).

### 4.5 Aspects for improvement

In this study, both the importance and ratings of nurse communication and registration communication were comparatively lower than those of high-quality doctor communication (Figure 1, 2). Consequently, improving the communication skills of all clinical staff, including nurses and registration personnel, emerges as an effective strategy for enhancing patient satisfaction (56–60).

As another primary factor influencing outpatient satisfaction, the environmental factor received low scores from all patients, especially from older adults (age over 80) and non-local patients (rural medical insurance) (61) [Figure 4, Age (Group)]. The lowest satisfaction score for layout also suggests a need for improvement in simplifying the hospital layout, particularly for first-time visitors (62).

Medical staff, particularly in departments with higher patient volumes, faced challenges maintaining a satisfactory environment due to patient overload (63, 64) (Figure 4, Department you registered). Employing telemedicine as a means to alleviate the volume of outpatient visits could prove to be an effective strategy (65).

### 4.6 Limitations

This survey faces two types of selection bias. First, data collection spanned the entire COVID-19 pandemic spectrum in China, from “total lockdown” (April) to “complete opening” (July), primarily capturing responses from “non-target” participants, particularly those undergoing PCR tests, potentially influencing the hospital’s general satisfaction level negatively due to lower satisfaction among these patients.

Second, the survey was distributed to all registered outpatients via messages (SMS), potentially attracting a higher proportion of participants with advanced educational backgrounds or familiarity with online surveys. The higher education bias, where individuals with higher education tend to give lower satisfaction assessments, likely contributed to an overall decrease in satisfaction levels.

Moreover, this study was carried out in a single hospital; therefore, the findings are not representative of the whole of China. As a cross-sectional survey, no causal inferences could be made. Considering the abovementioned limitations, the results may not be generalized to depict the patients’ satisfaction level in other healthcare facilities in China.

## 5 Conclusion

The national standard questionnaire showed adaptability for satisfaction surveys in this tertiary hospital, revealing doctor communication and environment as primary factors influencing outpatient satisfaction. Satisfaction levels, assessed higher than national and regional averages, varied based on socio-demographic characteristics. Patient overload emerged as a notable issue affecting satisfaction, suggesting areas for improvement, such as enhancing communication skills, simplifying medical routes, and addressing patient volume.

## Data availability statement

The data analyzed in this study is subject to the following licenses/restrictions: the data supporting the findings of this study are accessible from Shenyang the Fourth People’s Hospital, but access to these data is restricted due to licensing agreements for the current study, and therefore, they are not publicly available. However, interested parties may obtain the data from the authors upon reasonable request, subject to permission from Shenyang the Fourth People’s Hospital. Requests to access these datasets should be directed to XIN_TAO163@163.COM.

## Ethics statement

The studies involving humans were approved by Institutional Review Board (IRB) of Shenyang Fourth People’s Hospital. The studies were conducted in accordance with the local legislation and institutional requirements. Written informed consent for participation in this study was provided by the participants’ legal guardians/next of kin.

## Author contributions

ZX: Data curation, Formal analysis, Funding acquisition, Project administration, Writing – original draft. XT: Data curation, Resources, Writing – review & editing. WS: Project administration, Resources, Writing – review & editing. KH: Writing – review & editing. GH: Writing – review & editing. MM: Methodology, Supervision, Writing – review & editing.
