# Corrigendum: An evaluation of outpatient satisfaction based on the national standard questionnaire: a satisfaction survey conducted in a tertiary hospital in Shenyang, China

**DOI:** 10.3389/fpubh.2024.1503673

**Published:** 2024-10-15

**Authors:** Zhou Xintong, Xin Tao, Wang Shuying, K. A. T. M. Ehsanul Huq, Gao Huiying, Moriyama Michiko

**Affiliations:** ^1^Department of Hospital Infection Management, Shenyang the Fourth People's Hospital, Shenyang, China; ^2^Graduate School of Biomedical and Health Sciences, Division of Integrated Health Sciences, Hiroshima University, Hiroshima, Japan; ^3^Department of Information, Shenyang the Fourth People's Hospital, Shenyang, China; ^4^Department of Doctor-patient Communication, Shenyang the Fourth People's Hospital, Shenyang, China

**Keywords:** patient satisfaction, healthcare survey, doctor–patient relationship, environment, factor analysis

In the published article, there was an error in affiliations [1, 2]. Instead of “[^1^Graduate School of Biomedical and Health Sciences, Division of Integrated Health Sciences, Hiroshima University, Hiroshima, Japan, ^2^Department of Hospital Infection Management, Shenyang the Fourth People's Hospital, Shenyang, China]”, it should be “[^1^Department of Hospital Infection Management, Shenyang the Fourth People's Hospital, Shenyang, China, ^2^Graduate School of Biomedical and Health Sciences, Division of Integrated Health Sciences, Hiroshima University, Hiroshima, Japan]”.

The authors apologize for this error and state that this does not change the scientific conclusions of the article in any way. The original article has been updated.

