# Supplementary material for: An evaluation of outpatient satisfaction based on the national standard questionnaire: a satisfaction survey conducted in a tertiary hospital in Shenyang, China
Source: Front Public Health. 2024 May 9;12:1348426. doi: 10.3389/fpubh.2024.1348426 (PMC11111912; doi:10.3389/fpubh.2024.1348426)
Supplement: Supplementary file 1 [file Table_1.DOCX]

| ***Outpatient Satisfaction Questionnaire*** | | | | | | | | | | | | | | | | | | | | | | | | | | | | |
| --- | --- | --- | --- | --- | --- | --- | --- | --- | --- | --- | --- | --- | --- | --- | --- | --- | --- | --- | --- | --- | --- | --- | --- | --- | --- | --- | --- | --- |
| **Dimensions and Values** | | | | | | | | | | | | | | | | | | | | | | | | | | | | |
| 1. How did you registered? | | | | | | | | | | | | | | | | | | | | | | | | | | | | |
| On Site = 1 | | | Appointment = 2 | | | | | | | | | | Self-service Machine = 3 | | | | | | | Others = 4 | | | | | |  | | |
| **Convenience** | | | | | | | | | | | | | | | | | | | | | | | | | | | | |
| 2. If you registered on site, how many minutes have you waited in the queue? | | | | | | | | | | | | | | | | | | | | Open Blank | | | | | | | | |
| 3. Do you think the registration is convenient? | | | | | | | | | | | | | | | | | | | | | | | | | | | | |
| Very inconvenient = 1 | | | A bit inconvenient = 2 | | | | | | | | | | Relatively convenient = 3 | | | | | | | | Very convenient = 4 | | | | | | I don't know = 0 | |
| **Registration Communication** | | | | | | | | | | | | | | | | | | | | | | | | | | | | |
| 4. Did the registration staff respect you? | | | | | | | | | | | | | | | | | | | | | | | | | | | | |
| Very disrespectful = 1 | | | A bit disrespectful = 2 | | | | | | | | | | Relatively respectful = 3 | | | | | | | | Very respectful = 4 | | | | | | I don't know = 0 | |
| 5. Did the registration staff listen to you carefully? | | | | | | | | | | | | | | | | | | | | | | | | | | | | |
| Not carefully at all = 1 | | | A bit not carefully = 2 | | | | | | | | | | Relatively carefully = 3 | | | | | | | | Very carefully = 4 | | | | |  | | |
| **Doctor Communication** | | | | | | | | | | | | | | | | | | | | | | | | | | | | |
| 6. Did the doctors respect you? | | | | | | | | | | | | | | | | | | | | | | | | | | | | |
| Very disrespectful = 1 | | | A bit disrespectful = 2 | | | | | | | | | | Relatively respectful = 3 | | | | | | | | Very respectful = 4 | | | | | |  | |
| 7. Did the doctors listen to you carefully? | | | | | | | | | | | | | | | | | | | | | | | | | | | | |
| Not carefully at all = 1 | | | A bit not carefully = 2 | | | | | | | | | | Relatively carefully = 3 | | | | | | | | Very carefully = 4 | | | | |  | | |
| 8. Can you understand the doctors' explains? | | | | | | | | | | | | | | | | | | | | | | | | | | | | |
| Can't understand at all = 1 | | | | | A bit can't understand = 2 | | | | | | | | | | Basically understood = 3 | | | | | | | | | Perfectly understood = 4 | | | | |
| **Nurse Communication** | | | | | | | | | | | | | | | | | | | | | | | | | | | | |
| 9. Did the nurses respect you? | | | | | | | | | | | | | | | | | | | | | | | | | | | | |
| Very disrespectful = 1 | | A bit disrespectful = 2 | | | | | | | | | | Relatively respectful = 3 | | | | | | Very respectful = 4 | | | | | | | I haven't seen a nurse = 0 | | | |
| 10. Did the nurses listen to you carefully? | | | | | | | | | | | | | | | | | | | | | | | | | | | | |
| Not carefully at all = 1 | | | | A bit not carefully = 2 | | | | | | | | | Relatively carefully = 3 | | | | | | Very carefully = 4 | | | | | | |  | | |
| 11. Can you understand the nurses' explains? | | | | | | | | | | | | | | | | | | | | | | | | | | | | |
| Can't understand at all = 1 | | | | | | A bit can't understand = 2 | | | | | | | | | Basically understood = 3 | | | | | | | | | Perfectly understood = 4 | | | | |
| **Environment** | | | | | | | | | | | | | | | | | | | | | | | | | | | | |
| 12. Are the road signs and instructions in the hospital clear? | | | | | | | | | | | | | | | | | | | | | | | | | | | | |
| Very unclear = 1 | | | A bit unclear = 2 | | | | | | | | | | Relatively clear = 3 | | | | | | | | Very clear = 4 | | | | |  | | |
| 13. What is your general impression of the hospital's facilities? (Such as seats, elevators, drinking water machines, etc.) | | | | | | | | | | | | | | | | | | | | | | | | | | | | |
| Very dissatisfied = 1 | | | A bit dissatisfied = 2 | | | | | | | | | | Relatively satisfied = 3 | | | | | | | | Very satisfied = 4 | | | | |  | | |
| 14. Was the toilet clean? | | | | | | | | | | | | | | | | | | | | | | | | | | | | |
| Not clean at all = 1 | | | A bit not clean = 2 | | | | | | | | | | Relatively clean = 3 | | | | | | | | Very clean = 4 | | | | | I never used it = 0 | | |
| 15. Is the spatial distribution of the hospital convenient? (Floors and distance of the departments such as the triage, registration, consulting room, examination room, cashier, etc.) | | | | | | | | | | | | | | | | | | | | | | | | | | | | |
| Very inconvenient = 1 | | | | A bit inconvenient = 2 | | | | | | | | | Relatively convenient = 3 | | | | | | | | Very convenient = 4 | | | | | |  | |
| **Respond of Needs** | | | | | | | | | | | | | | | | | | | | | | | | | | | | |
| 16. During the diagnosis and treatment process, did the medical staff pay attention to protecting your privacy? (e.g.If the curtain is pulled up during the inspection?) | | | | | | | | | | | | | | | | | | | | | | | | | | | | |
| Didn't pay attention at all = 1 | | | | | | | | | Paid little attention = 2 | | | | | | Paid some attention = 3 | | | | | | | | | Paid full attention = 4 | | | | |
| 17. Can your complaints and dissatisfaction during the medical treatment process be responded to in a timely manner? | | | | | | | | | | | | | | | | | | | | | | | | | | | | |
| Not timely at all = 1 | Basically not timely = 2 | | | | | | | | | | Basically timely = 3 | | | | | | Very timely = 4 | | | | | I have no complains or dissatisfaction = 0 | | | | | | |
| **General Satisfaction Level** | | | | | | | | | | | | | | | | | | | | | | | | | | | | |
| 1. Which number best represents your general evaluation of this hospital?   (1 being worst, 10 being best) | | | | | | | | | | | | | | | | | | | | | Open Blank (Integers from 1 to 10) | | | | | | | |
| 19. Would you recommend this hospital to relatives and friends? | | | | | | | | | | | | | | | | | | | | | | | | | | | | |
| Certainly not = 1 | | | Probably not = 2 | | | | | | | | | | Probably will = 3 | | | | | | | | Certainly will = 4 | | | | |  | | |
| 20. Department you registered (If there are more than one, please fill in the first one) | | | | | | | | | | | | | | | | | | | | | | | | | | | | |
| Internal Medicine = 1 | | | Surgical = 2 | | | | | | | Ophthalmology = 3 | | | | | | Obstetric & Pediatric = 4 | | | | | | | COVID-19 PCR = 5 | | | | | Others =6 |
| 21. Type of your registration | | | | | | | | | | | | | | | | | | | | | | | | | | | | |
| Normal = 1 | | | Special = 2 | | | | | | | | | |  | | | | | | | |  | | | | |  | | |
| 22. Gender | | | | | | | | | | | | | | | | | | | | | | | | | | | | |
| Male = 1 | | | Female = 2 | | | | | | | | | |  | | | | | | | |  | | | | |  | | |
| 23. Age (Group) | | | | | | | | | | | | | | | | | | | | | | | | | | | | |
| Under 20 = 1 | | | 20 to 39 = 2 | | | | | | | | | | 40 to 59 = 3 | | | | | | | | 60 to 79 = 4 | | | | | 80 and above = 5 | | |
| 24. Education Background | | | | | | | | | | | | | | | | | | | | | | | | | | | | |
| Middle school or lower = 1 | | | | | | | High school = 2 | | | | | | | Undergraduate = 3 | | | | | | | Graduate = 4 | | | | |  | | |
| 25. Payment Method | | | | | | | | | | | | | | | | | | | | | | | | | | | | |
| Free medical insurance = 1 | | | | | | | | Rural insurance = 2 | | | | | | Urban insurance = 3 | | | | | | | Self-payment = 4 | | | | |  | | |
