# Supplementary material for: An evaluation of outpatient satisfaction based on the national standard questionnaire: a satisfaction survey conducted in a tertiary hospital in Shenyang, China
Source: Front Public Health. 2024 May 9;12:1348426. doi: 10.3389/fpubh.2024.1348426 (PMC11111912; doi:10.3389/fpubh.2024.1348426)
Supplement: Supplementary file 2 [file Table_2.DOCX]

| ***Statistical description of satisfaction items*** | | | |  |
| --- | --- | --- | --- | --- |
| **Factors and items** | **Count** | **Percentage** | **Valid percentage** | **M (SD) ^c^** |
| *Convenience* |  |  |  |  |
| “ convenience of registration” |  |  |  |  |
| Very inconvenient = 1 | 386 | 6.4% | 6.5% | 3.42 (0.83) |
| A bit inconvenient = 2 | 166 | 2.8% | 2.8% |  |
| Relatively convenient = 3 | 1955 | 32.5% | 32.7% |  |
| Very convenient = 4 | 3471 | 57.7% | 58.1% |  |
| I don't know = 0 | 34 | 0.6% | — |  |
| *Registration communication* |  |  |  |  |
| “respect from registration staff” |  |  |  |  |
| Very disrespectful = 1 | 125 | 2.1% | 2.8% | 3.46 (0.68) |
| A bit disrespectful = 2 | 98 | 1.6% | 2.2% |  |
| Relatively respectful = 3 | 1822 | 30.3% | 41.4% |  |
| Very respectful = 4 | 2360 | 39.3% | 53.6% |  |
| I don't know = 0 ^a^ | 1607 | 26.7% | — |  |
| “attentiveness of registration staff” |  |  |  |  |
| Not carefully at all = 1 | 96 | 1.6% | 2.2% | 3.38 (0.66) |
| A bit not carefully = 2 | 144 | 2.4% | 3.3% |  |
| Relatively carefully = 3 | 2167 | 36.0% | 49.2% |  |
| Very carefully = 4 | 1998 | 33.2% | 45.4% |  |
| Automatically skipped ^a^ | 1607 | 26.7% | — |  |
| *Doctor communication* |  |  |  |  |
| “respect from doctors” |  |  |  |  |
| Very disrespectful = 1 | 140 | 2.3% | 2.3% | 3.61 (0.66) |
| A bit disrespectful = 2 | 162 | 2.7% | 2.7% |  |
| Relatively respectful = 3 | 1622 | 27.0% | 27.0% |  |
| Very respectful = 4 | 4088 | 68.0% | 68.0% |  |
| “attentiveness of doctors” |  |  |  |  |
| Not carefully at all = 1 | 118 | 2.0% | 2.0% | 3.61 (0.65) |
| A bit not carefully = 2 | 215 | 3.6% | 3.6% |  |
| Relatively carefully = 3 | 1588 | 26.4% | 26.4% |  |
| Very carefully = 4 | 4091 | 68.0% | 68.0% |  |
| “clarity of doctor's explanations” |  |  |  |  |
| Can't understand at all = 1 | 73 | 1.2% | 1.2% | 3.69 (0.57) |
| A bit can't understand = 2 | 104 | 1.7% | 1.7% |  |
| Basically understood = 3 | 1456 | 24.2% | 24.2% |  |
| Perfectly understood = 4 | 4379 | 72.8% | 72.8% |  |
| *Nurse communication* |  |  |  |  |
| “respect from nurses” |  |  |  |  |
| Very disrespectful = 1 | 92 | 1.5% | 2.0% | 3.54 (0.65) |
| A bit disrespectful = 2 | 144 | 2.4% | 3.1% |  |
| Relatively respectful = 3 | 1602 | 26.6% | 34.0% |  |
| Very respectful = 4 | 2875 | 47.8% | 61.0% |  |
| I haven't seen a nurse = 0 ^b^ | 1299 | 21.6% | — |  |
| “attentiveness of nurses” |  |  |  |  |
| Not carefully at all = 1 | 83 | 1.4% | 1.8% | 3.46 (0.66) |
| A bit not carefully = 2 | 184 | 3.1% | 3.9% |  |
| Relatively carefully = 3 | 1935 | 32.2% | 41.1% |  |
| Very carefully = 4 | 2511 | 41.8% | 53.3% |  |
| Automatically skipped ^b^ | 1299 | 21.6% | — |  |
| “clarity of nurse's explanations” |  |  |  |  |
| Can't understand at all = 1 | 63 | 1.0% | 1.3% | 3.60 (0.59) |
| A bit can't understand = 2 | 74 | 1.2% | 1.6% |  |
| Basically understood = 3 | 1540 | 25.6% | 32.7% |  |
| Perfectly understood = 4 | 3036 | 50.5% | 64.4% |  |
| Automatically skipped ^b^ | 1299 | 21.6% | — |  |
| *Environment* |  |  |  |  |
| “signs and instruction” |  |  |  |  |
| Very unclear = 1 | 103 | 1.7% | 1.7% | 3.41 (0.66) |
| A bit unclear = 2 | 291 | 4.8% | 4.8% |  |
| Relatively clear = 3 | 2650 | 44.1% | 44.1% |  |
| Very clear = 4 | 2968 | 49.4% | 49.4% |  |
| “facilities” |  |  |  |  |
| Very dissatisfied = 1 | 102 | 1.7% | 1.7% | 3.35 (0.66) |
| A bit dissatisfied = 2 | 312 | 5.2% | 5.2% |  |
| Relatively satisfied = 3 | 2972 | 49.4% | 49.4% |  |
| Very satisfied = 4 | 2626 | 43.7% | 43.7% |  |
| “toilet” |  |  |  |  |
| Not clean at all = 1 | 44 | 0.7% | 1.0% | 3.43 (0.62) |
| A bit not clean = 2 | 171 | 2.8% | 4.1% |  |
| Relatively clean = 3 | 1920 | 31.9% | 45.7% |  |
| Very clean = 4 | 2069 | 34.4% | 49.2% |  |
| I never used it = 0 | 1808 | 30.1% | — |  |
| “layout” |  |  |  |  |
| Very inconvenient = 1 | 114 | 1.9% | 1.9% | 3.24 (0.65) |
| A bit inconvenient = 2 | 374 | 6.2% | 6.2% |  |
| Relatively convenient = 3 | 3504 | 58.3% | 58.3% |  |
| Very convenient = 4 | 2020 | 33.6% | 33.6% |  |
| *Response of needs* | | |  |  |
| “privacy” | | |  |  |
| Didn't pay attention at all = 1 | 55 | 0.9% | 0.9% | 3.45 (0.61) |
| Paid little attention = 2 | 202 | 3.4% | 3.4% |  |
| Paid some attention = 3 | 2751 | 45.8% | 45.8% |  |
| Paid full attention = 4 | 3004 | 50.0% | 50.0% |  |
| “respond of complain” | | |  |  |
| Not timely at all = 1 | 202 | 3.4% | 7.7% | 3.14 (0.9) |
| Basically not timely = 2 | 290 | 4.8% | 11.1% |  |
| Basically timely = 3 | 1047 | 17.4% | 40.1% |  |
| Very timely = 4 | 1070 | 17.8% | 41.0% |  |
| I have no complains or dissatisfaction = 0 | 3403 | 56.6% | — |  |
| *General satisfaction indicators* |  |  |  |  |
| “patient’s overall evaluation of this hospital” (overall evaluation) |  |  |  |  |
| 1 | 118 | 2.0% | 2.0% | 8.43 (1.97) |
| 2 | 49 | 0.8% | 0.8% |  |
| 3 | 79 | 1.3% | 1.3% |  |
| 4 | 81 | 1.3% | 1.3% |  |
| 5 | 221 | 3.7% | 3.7% |  |
| 6 | 216 | 3.6% | 3.6% |  |
| 7 | 384 | 6.4% | 6.4% |  |
| 8 | 1210 | 20.1% | 20.1% |  |
| 9 | 1401 | 23.3% | 23.3% |  |
| 10 | 2253 | 37.5% | 37.5% |  |
| “recommendation of this hospital to others” (recommendation level) | |  |  |  |
| Certainly not = 1 | 107 | 1.8% | 1.8% | 3.54 (0.64) |
| Probably not = 2 | 175 | 2.9% | 2.9% |  |
| Probably will = 3 | 2087 | 34.7% | 34.7% |  |
| Certainly will = 4 | 3643 | 60.6% | 60.6% |  |
| ^a^ If the answer of Q4 is "I don’t know", Q5 will be automatically skipped | | | |  |
| ^b^ If the answer of Q9 is "I haven’t seen a nurse", Q10 and Q11 will be automatically skipped | | | |  |
| ^C^ The M (SD) calculation excluded all options with a "0" value | | | |  |
