# Supplementary material for: An evaluation of outpatient satisfaction based on the national standard questionnaire: a satisfaction survey conducted in a tertiary hospital in Shenyang, China
Source: Front Public Health. 2024 May 9;12:1348426. doi: 10.3389/fpubh.2024.1348426 (PMC11111912; doi:10.3389/fpubh.2024.1348426)
Supplement: Supplementary file 3 [file Table_3.DOCX]

*supplementary 3*

Corresponding percentage of the means of general satisfaction indicators. (A) Month; (B) Registration method; (C) Department you registered; (D) Type of your registration; (E) Gender; (F) Age (Group); (G) Education background; (H) Payment method *P<0.05,***P<0.001


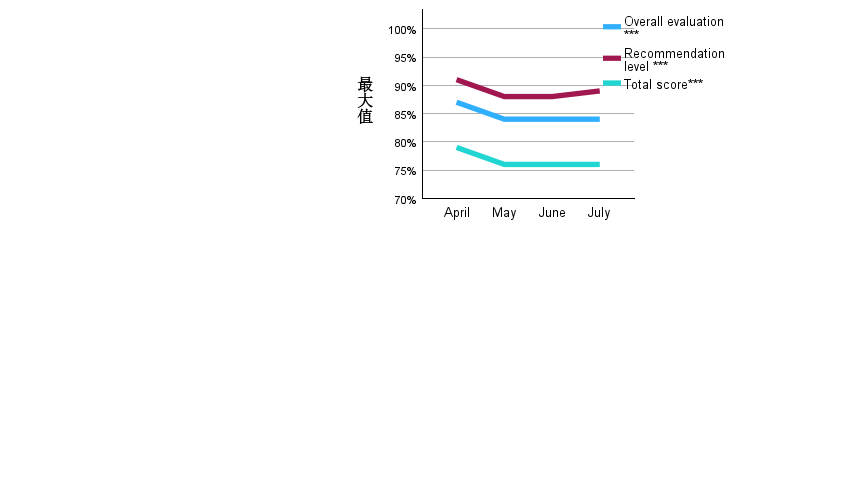

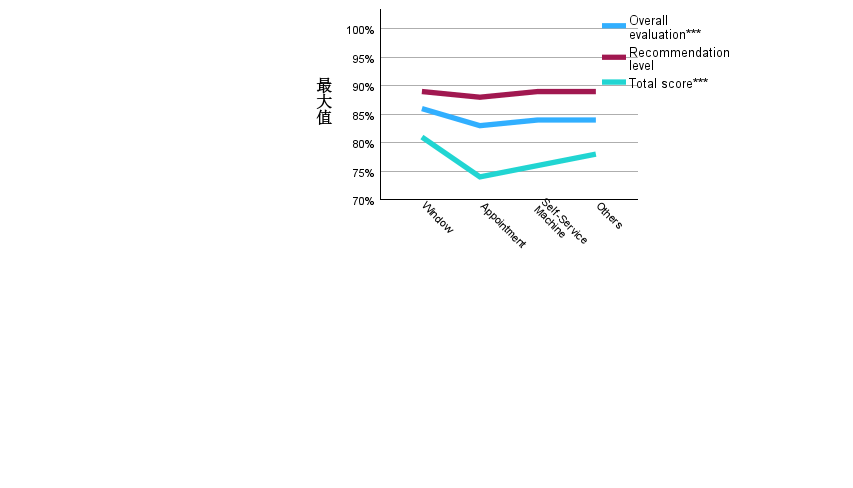

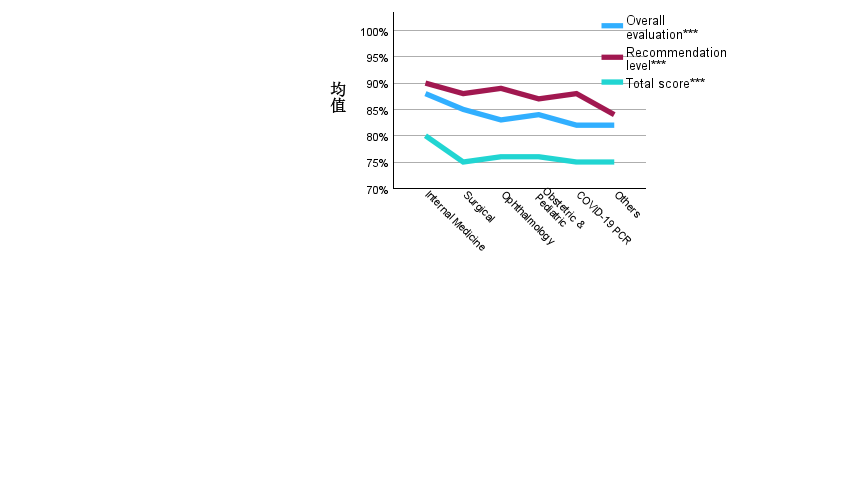

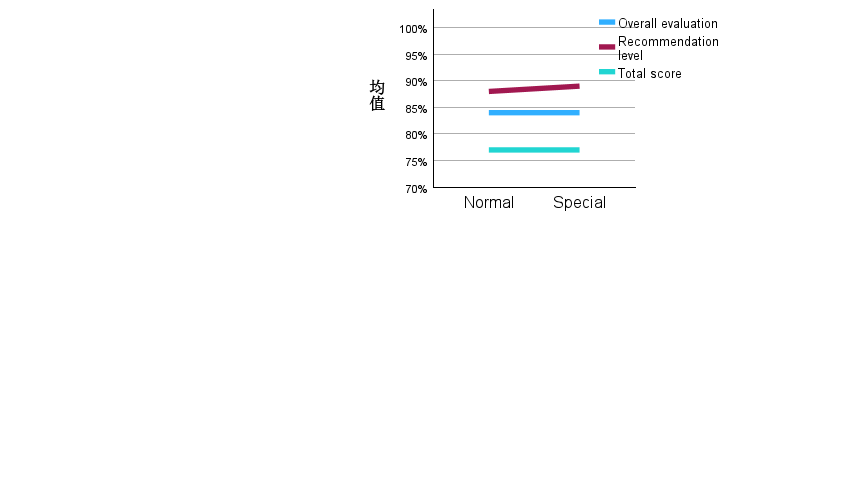

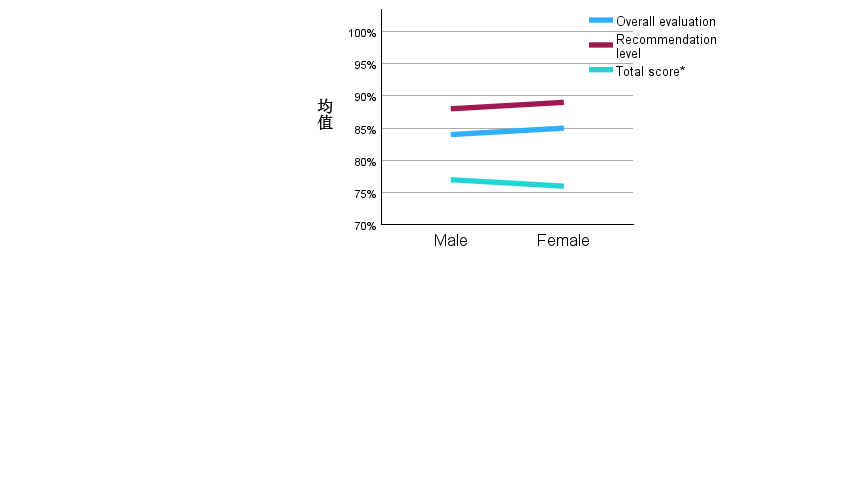

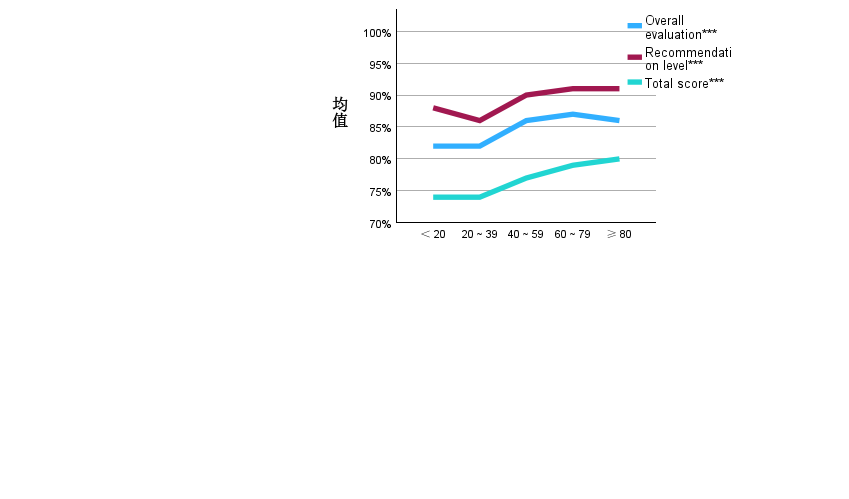

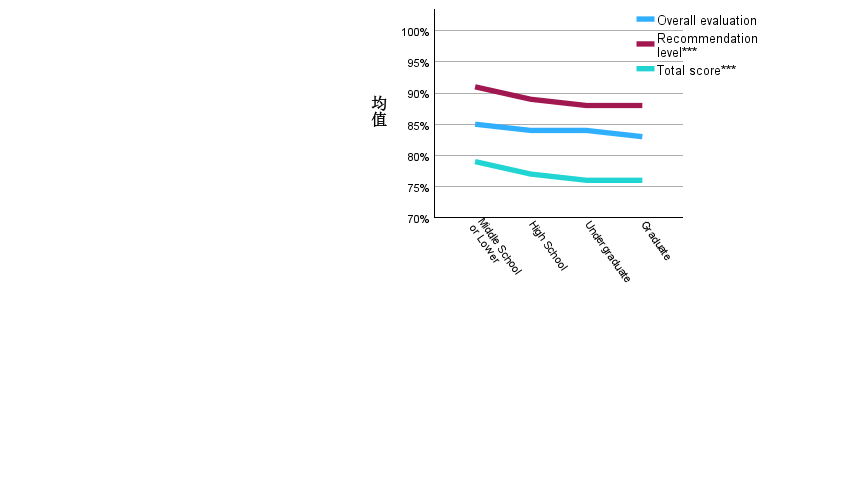

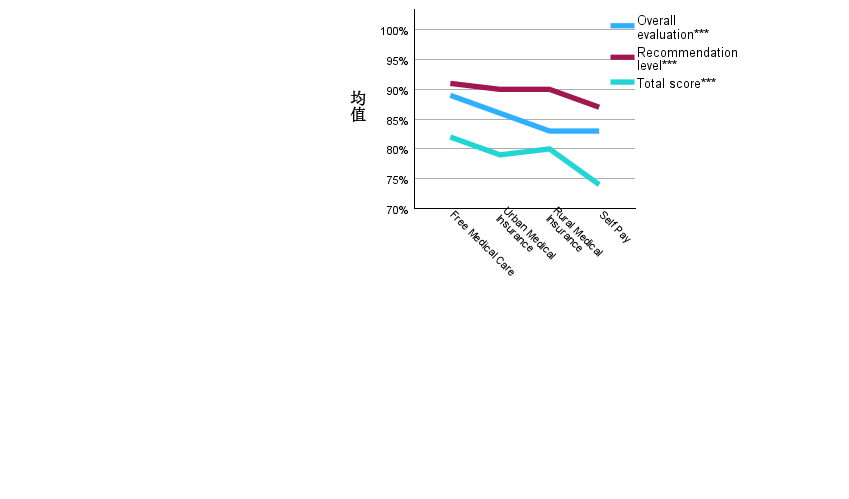


A

B

C

D

E

F

G

H
