# Supplementary material for: An evaluation of outpatient satisfaction based on the national standard questionnaire: a satisfaction survey conducted in a tertiary hospital in Shenyang, China
Source: Front Public Health. 2024 May 9;12:1348426. doi: 10.3389/fpubh.2024.1348426 (PMC11111912; doi:10.3389/fpubh.2024.1348426)
Supplement: Supplementary file 4 [file Table_4.DOCX]

*supplementary 4*


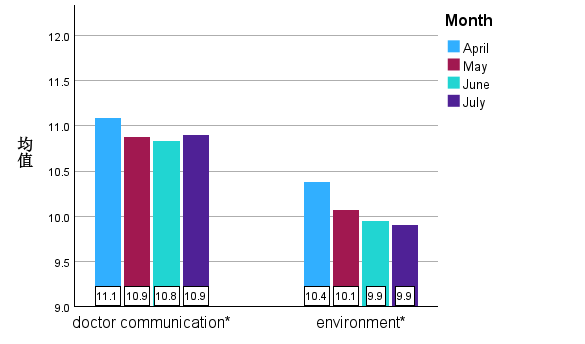

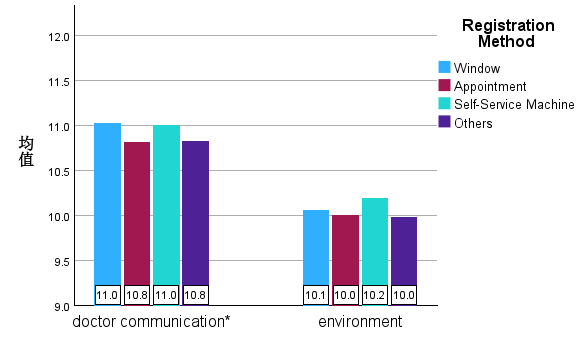

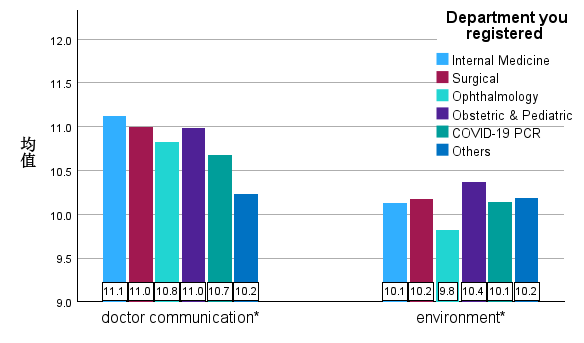

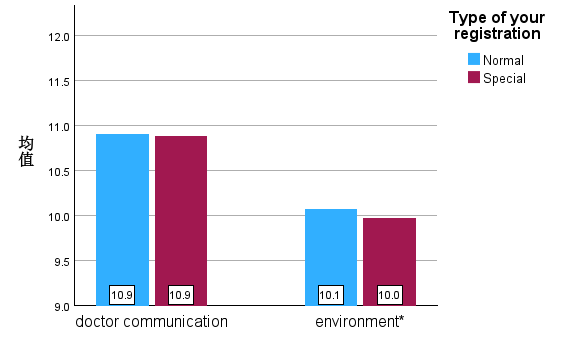

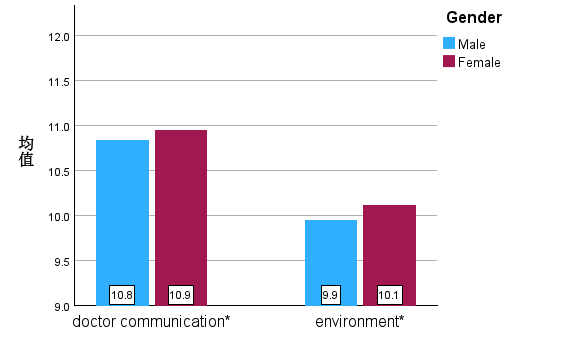

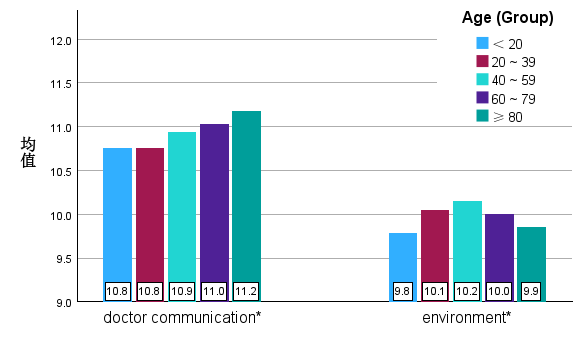

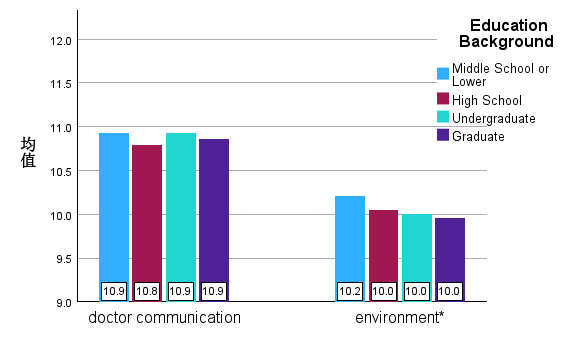

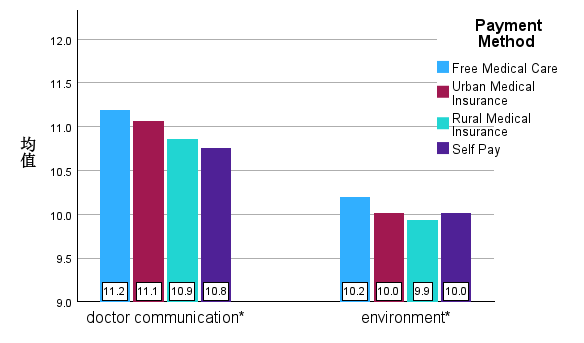


A

B

C

D

E

F

G

H

Comparison of the sum scores of the two primary factors. (A) Month; (B) Registration method; (C) Department you registered; (D) Type of your registration; (E) Gender; (F) Age (Group); (G) Education background; (H) Payment method *P<0.05,***P<0.001
