# Supplementary material for: An evaluation of outpatient satisfaction based on the national standard questionnaire: a satisfaction survey conducted in a tertiary hospital in Shenyang, China
Source: Front Public Health. 2024 May 9;12:1348426. doi: 10.3389/fpubh.2024.1348426 (PMC11111912; doi:10.3389/fpubh.2024.1348426)
Supplement: Supplementary file 5 [file Table_5.DOCX]

STROBE Statement—checklist of items included in reports of observational study

|  | Item No. | Recommendation | Page  No. | Relevant text from manuscript |
| --- | --- | --- | --- | --- |
| **Title and abstract** | 1 | (*a*) Indicate the study’s design with a commonly used term in the title or the abstract | 1 | Evaluation of Outpatient Satisfaction Based on National Standard Questionnaire: A Satisfaction Survey Conducted in a Tertiary Hospital in Shenyang, China |
|  |  | (*b*) Provide in the abstract an informative and balanced summary of what was done and what was found | 1 | Background, Method, Results,Conclusion |
| Introduction | | | |  |
| Background/rationale | 2 | Explain the scientific background and rationale for the investigation being reported | 2 | 1 Introduction  Patient satisfaction survey serves as a pivotal tool in evaluating the quality of healthcare services (1, 2). Healthcare institutions can address issues and areas of improvement by getting feedback from the patients (3), enabling targeted enhancements to elevate the overall quality of healthcare services (4, 5). Additionally, this survey creates a platform for patients to voice their opinions and offer suggestions, fostering positive communication between patients and healthcare providers (6). This dynamic fostering the provision of positive doctor-patient relationships (7)... |
| Objectives | 3 | State specific objectives, including any prespecified hypotheses | 3 | 1 Introduction  Hence, it is necessary to implement the national version questionnaire in a satisfaction survey conducted by an independent medical facility for two primary reasons: 1) to validate the suitability and adaptability of this standardized questionnaire, and 2) to evaluate the satisfaction levels of individual health care facility using this standardized measurement and address main aspects for improvement. |
| Methods | | | |  |
| Study design | 4 | Present key elements of study design early in the paper | 3 | 2.1 Study design and setting |
| Setting | 5 | Describe the setting, locations, and relevant dates, including periods of recruitment, exposure, follow-up, and data collection | 3 | 2.1 Study design and setting  2.2 Participants and study procedures |
| Participants | 6 | (*a*) *Cohort study*—Give the eligibility criteria, and the sources and methods of selection of participants. Describe methods of follow-up  *Case-control study*—Give the eligibility criteria, and the sources and methods of case ascertainment and control selection. Give the rationale for the choice of cases and controls  *Cross-sectional study*—Give the eligibility criteria, and the sources and methods of selection of participants | 3-4 | 2.2 Participants and study procedures  The questionnaire used in this study was distributed to all registered patients via text message links from April to July, 2022. |
|  |  | (*b*) *Cohort study*—For matched studies, give matching criteria and number of exposed and unexposed  *Case-control study*—For matched studies, give matching criteria and the number of controls per case |  | Not applicable. |
| Variables | 7 | Clearly define all outcomes, exposures, predictors, potential confounders, and effect modifiers. Give diagnostic criteria, if applicable | 4 | 2.3 Measuring tool |
| Data sources/ measurement | 8* | For each variable of interest, give sources of data and details of methods of assessment (measurement). Describe comparability of assessment methods if there is more than one group | 4-5 | 2.4 Statistical Analysis |
| Bias | 9 | Describe any efforts to address potential sources of bias | 4 | 2.4.1Data preparation  a total of 1,777 invalid replies out of 7,789 (22.8%) were excluded due to duplicate or contradictory answer (consistency bias) |
| Study size | 10 | Explain how the study size was arrived at | 4 | 2.4.1Data preparation  A total of 7789 patients were responded from 402,964 registered as outpatients (1.93% responded) and a total of 1,777 invalid replies out of 7,789 (22.8%) were excluded due to duplicate or contradictory answer (consistency bias), and 6,012 valid replies were used for analyses. |

Continued on next page

| Quantitative variables | 11 | Explain how quantitative variables were handled in the analyses. If applicable, describe which groupings were chosen and why | 4-5 | 2.4.2 Data analysis |
| --- | --- | --- | --- | --- |
| Statistical methods | 12 | (*a*) Describe all statistical methods, including those used to control for confounding | 4 | 2.4.2 Data analysis  SPSS Statistics 28.0 and SPSS Amos 28.0 (IBM) were used in this study for descriptive analysis, comparative analysis and confirmatory factor analysis (CFA). |
|  |  | (*b*) Describe any methods used to examine subgroups and interactions | 5 | 2.4.2 Data analysis  The differences of general satisfaction level among outpatient’s demographic characteristics were assessed by t-test or analysis of variance. To compare the results of overall evaluation, recommendation level and total satisfaction score, their means were converted into corresponding percentages. Finally, the area’s most in need of improvement was allocated by comparing the items with the lowest means with other factors. The significance level was set at < 0.05. P values exceeding 0.05 are not listed or marked in the subsequent content. |
|  |  | (*c*) Explain how missing data were addressed | 4 | 2.4.1 Data preparation a total of 1,777 invalid replies out of 7,789 (22.8%) were excluded due to duplicate or contradictory answer (consistency bias) |
|  |  | (*d*) *Cohort study*—If applicable, explain how loss to follow-up was addressed  *Case-control study*—If applicable, explain how matching of cases and controls was addressed  *Cross-sectional study*—If applicable, describe analytical methods taking account of sampling strategy |  | Not applicable. |
|  |  | (*e*) Describe any sensitivity analyses |  | Not applicable. |
| Results | | | | |
| Participants | 13* | (a) Report numbers of individuals at each stage of study—eg numbers potentially eligible, examined for eligibility, confirmed eligible, included in the study, completing follow-up, and analysed | 4 | 2.4.1Data preparation  A total of 7789 patients were responded from 402,964 registered as outpatients (1.93% responded) and a total of 1,777 invalid replies out of 7,789 (22.8%) were excluded due to duplicate or contradictory answer (consistency bias), and 6,012 valid replies were used for analyses. |
|  |  | (b) Give reasons for non-participation at each stage | 3 | 2.2 Participants and study procedures  Patients voluntarily decided whether to participate in the survey. |
|  |  | (c) Consider use of a flow diagram |  | Not applicable. |
| Descriptive data | 14* | (a) Give characteristics of study participants (eg demographic, clinical, social) and information on exposures and potential confounders | 5 | 3.1 Socio-demographic characteristics of patients |
|  |  | (b) Indicate number of participants with missing data for each variable of interest | 20 | Table 1 |
|  |  | (c) *Cohort study*—Summarise follow-up time (eg, average and total amount) |  | Not applicable. |
| Outcome data | 15* | *Cohort study*—Report numbers of outcome events or summary measures over time |  | Not applicable. |
|  |  | *Case-control study—*Report numbers in each exposure category, or summary measures of exposure |  | Not applicable. |
|  |  | *Cross-sectional study—*Report numbers of outcome events or summary measures | 5 | 3.2 Model fit analysis: confirmatory factor analysis (CFA), Figure 1, Table 2 |
| Main results | 16 | (*a*) Give unadjusted estimates and, if applicable, confounder-adjusted estimates and their precision (eg, 95% confidence interval). Make clear which confounders were adjusted for and why they were included | 5-6 | 3.2.1Validity and reliability  3.2.2CFA model fit  3.3 Satisfaction level of outpatients,Figure 2 |
|  |  | (*b*) Report category boundaries when continuous variables were categorized |  | Not applicable. |
|  |  | (*c*) If relevant, consider translating estimates of relative risk into absolute risk for a meaningful time period |  | Not applicable. |

Continued on next page

| Other analyses | 17 | Report other analyses done—eg analyses of subgroups and interactions, and sensitivity analyses | 6-7, 21-22 | 3.4 Analyzing influence of patient’s socio-demographics on general satisfaction level  3.5 Analyzing primary factors which affect patients’ general satisfaction levels  3.6 Investigating influencing factors on primary factors  Figure 3, Figure 4, Table 3 |
| --- | --- | --- | --- | --- |
| Discussion | | | | |
| Key results | 18 | Summarise key results with reference to study objectives | 7-10 | 4 Discussion  This marks the inaugural publication detailing the outcomes of a national satisfaction survey conducted at a tertiary hospital in China. Through rigorous examinations of validity and reliability, we ascertained that the survey consistently adhered to established questionnaire structure. The survey’s comprehensive approach effectively identified six influencing factors in outpatient satisfaction: doctor communication, environment, registration method, age (group), department of registration, and payment method. This conclusion is supported by a substantial volume of outpatient data... |
| Limitations | 19 | Discuss limitations of the study, taking into account sources of potential bias or imprecision. Discuss both direction and magnitude of any potential bias | 10 | 4.6 Limitation  This survey faces two types of selection bias... |
| Interpretation | 20 | Give a cautious overall interpretation of results considering objectives, limitations, multiplicity of analyses, results from similar studies, and other relevant evidence |  | 4.1 Questionnaire adaptability and its influences  4.2 Satisfaction level and influencing factors  4.3 The Impact of Unique Chinese Factors on Patient Satisfaction  4.4 Gaps between patients’ expectations and actual experience |
| Generalisability | 21 | Discuss the generalisability (external validity) of the study results |  | 4.2 Satisfaction level and influencing factors  This study reveals that the hospital has exceeded both national and northeast regional averages in scores for overall assessment and recommendation level (16). The significant factor contributing to this achievement is the hospital’s outstanding performance in the realm of doctor communication, particularly in the facet of clarity of doctor’s explanations, where it achieved the highest score (Figure 2). A wealth of research consistently supports the idea that thorough and effective communication by health care providers plays a pivotal role in significantly enhancing patient satisfaction levels (30, 32, 36, 37). |
| Other information | |  | | |
| Funding | 22 | Give the source of funding and the role of the funders for the present study and, if applicable, for the original study on which the present article is based |  | 9 Funding  This research received financial support from the 2022 Scientific Research Project of the Shenyang Health Commission (Project No. 2022021). |

*Give information separately for cases and controls in case-control studies and, if applicable, for exposed and unexposed groups in cohort and cross-sectional studies.
